# Prognostication of Follicular Lymphoma: A Review of Prognostic Scores and Factors

**DOI:** 10.3390/diagnostics15050647

**Published:** 2025-03-06

**Authors:** Ádám Jóna, Evelin Kiss, Árpád Illés

**Affiliations:** 1 Department of Hematology, Faculty of Medicine, University of Debrecen, Nagyerdei krt. 98, 4028 Debrecen, Hungary; evlnkiss92@gmail.com (E.K.); illesarpaddr@gmail.com (Á.I.); 2 Doctoral School of Clinical Medicine, University of Debrecen, 4028 Debrecen, Hungary

**Keywords:** follicular lymphoma, prognostication, FLIPI, FLIPI2, PRIMA-PI, m7-FLIPI, PET/CT, lymphocyte/monocyte ratio

## Abstract

Follicular lymphoma (FL) is an indolent, rarely curable B-cell malignancy with a heterogeneous clinical course. While generally treatable, FL is characterized by remissions and relapses, and its clinical presentation varies widely. Rituximab has revolutionized FL treatment, significantly improving overall survival over the past two decades. Risk assessment typically relies on histological grade, tumor burden, and the Follicular Lymphoma International Prognostic Index, which incorporates factors like age, hemoglobin level, and Ann Arbor stage. However, these indices have limitations in fully capturing the clinical variability of FL. Some patients experience indolent disease for extended periods without requiring treatment, while others present with aggressive forms resistant to standard therapies. This review examines various prognostic factors in FL, including the FLIPI, FLIPI2, PRIMA-PI, and m7-FLIPI. The FLIPI, based on five risk factors, stratifies patients into low-, intermediate-, and high-risk groups. The FLIPI2 incorporates beta2-microglobulin and the longest diameter of the largest involved node, offering improved prognostication. The PRIMA-PI, designed for patients receiving rituximab-containing regimens, uses beta2-microglobulin, bone marrow involvement, and the longest diameter of the largest involved node. The m7-FLIPI integrates mutational status with FLIPI2 parameters, further refining risk stratification. The review also discusses clinical parameters like maximum standardized uptake value on PET/CT and lymphocyte/monocyte ratio as prognostic factors. A high SUVmax and low lymphocyte/monocyte ratio identify high-risk patients. While FL remains incurable, advances in immunochemotherapy and targeted therapies have improved outcomes. This review provides a comprehensive overview of prognostic tools in FL, emphasizing the importance of risk stratification for personalized treatment strategies.

## 1. Introduction

Follicular lymphoma is a B-cell-derived, indolent, and rarely curable disease, yet it is readily treatable for the vast majority of patients. The disease course is characterized by remissions and relapses, although the clinical spectrum can be heterogeneous. The use of rituximab has revolutionized the treatment strategy, leading to a constantly improving overall survival trend for patients over the past two decades [1]. Risk assessment is typically based on histological grade, tumor burden, and the Follicular Lymphoma International Prognostic Index [2]. Despite the widespread application of these prognostic indices, they possess limitations in comprehensively capturing the clinical variability observed in follicular lymphoma. Some patients may exhibit an indolent disease course for prolonged periods without necessitating treatment, while others may present with more aggressive forms refractory to standard therapeutic approaches.

This review is focusing on further clinical and biological parameters that can affect the outcome of follicular lymphoma patients.

## 2. Clinical Parameters as Prognostic Factors in Follicular Lymphoma

### 2.1. Follicular Lymphoma International Prognostic Index

The development of the Follicular Lymphoma International Prognostic Index (FLIPI) involved a rigorous process to identify the most reliable and clinically relevant factors for predicting survival in patients with follicular lymphoma. They aimed to create a tool that was both statistically sound and easy for clinicians to use in everyday practice.

Initially a univariate analysis, it assessed the following 14 pretreatment characteristics: age, sex, Ann Arbor stage, cell type, bone marrow involvement, number of nodal areas involved, largest tumor diameter, hemoglobin level, serum lactate dehydrogenase (LDH) level, serum β2-microglobulin level, performance status, erythrocyte sedimentation rate (ESR), serum albumin level, and peripheral blood lymphocyte count. All but ESR and albumin were found to be significantly associated with overall survival. The Cox analysis ultimately included the following eight parameters: age, sex, Ann Arbor stage, bone marrow involvement, number of involved nodal areas, hemoglobin level, lymphocyte count, and serum LDH level. All eight were statistically significant in the multivariate model. However, to simplify the index for practical use, the final FLIPI only included five parameters: age ≥ 60 years, Ann Arbor stage III/IV, hemoglobin < 12 g/dL, number of nodal areas ≥ 4, and serum LDH > upper limit of normal [2].

### 2.2. Follicular Lymphoma International Prognostic Index 2

The development of the Follicular Lymphoma International Prognostic Index 2 (FLIPI2) [3] aimed to refine the existing FLIPI by incorporating new insights and biomarkers for more accurate prognostication in follicular lymphoma. They recognized the value of the original FLIPI and used its five factors as a starting point. The study utilized a larger and more diverse patient cohort than previous studies, increasing the statistical power and generalizability of their findings. This extensive dataset allowed for a more robust analysis and identification of subtle prognostic factors. Beyond the existing FLIPI parameters, the FLIPI2 study explored the inclusion of β2-microglobulin (B2M), a protein associated with lymphocyte activity and a potential marker of tumor burden and cell turnover. They considered different ways of incorporating B2M, including adding B2M to the existing FLIPI score, replacing LDH with B2M in the FLIPI or creating a new index with B2M and other potential factors. Ultimately, they found that simply replacing LDH with B2M yielded the most accurate and robust prognostic index, leading to the creation of FLIPI2. The other approaches, while exploring additional parameters, did not improve prognostication compared to the simpler substitution.

Both FLIPI and FLIPI2 consider age >60 years, Ann Arbor stage III/IV, hemoglobin <120 g/L, and number of nodal areas ≥4. The key difference is that FLIPI2 replaces serum LDH> normal (used in FLIPI) with B2M > normal. This change was made because B2M is considered a more specific and sensitive marker of tumor burden and cell turnover in follicular lymphoma, especially with modern treatments. FLIPI2 is thought to be more accurate and reliable for predicting patient outcomes and guiding treatment decisions.

A short communication [4] validated FLIPI2 using data from 498 follicular lymphoma patients diagnosed between 1980 and 2008 from the Fondazione Italiana Linfomi database. The study confirmed that FLIPI2 effectively stratifies patients into different risk groups, showing a significant difference in 5-year progression-free survival rates. The 5-year overall survival (OS) rate for the entire cohort was 79%, while the 5-year progression-free survival (PFS) for the entire cohort was 60%. The median PFS for the whole group was 3.7 years. However, the study emphasizes the importance of stratifying by risk groups using FLIPI2. The median PFS was not reached for the low-risk group, the median PFS was 6.8 years for the intermediate-risk group, and the median PFS was 2.1 years for the high-risk group.

### 2.3. PRIMA Prognostic Index

Bachy et al. [5] investigated a simplified scoring system for patients with de novo follicular lymphoma who are initially treated with R-CHOP (rituximab, cyclophosphamide, vincristine, prednisolon) immunochemotherapy. The authors aimed to create a simplified scoring system based on easily measurable factors like LDH and B2M levels. To develop the simplified scoring system, they first analyzed data from the PRIMA training cohort, a subset of patients from the PRIMA trial. Specifically, they looked at the relationship between elevated LDH and β2M levels (above the upper limit of normal) and the patients’ event-free survival. They found that patients with elevated LDH had a 5-year PFS rate of 50%, compared to 78% for those with normal LDH levels. Similarly, patients with elevated β2M had a 5-year PFS rate of 55%, compared to 75% for those with normal β2M levels. They then used this information to develop a simple scoring system based on these two factors.

To validate their findings, the researchers then tested their scoring system on a separate group of patients: the pooled FL2000 and MER validation cohort, which consisted of 479 patients. This cohort included patients from two independent trials. The patient characteristics (age, sex, stage, and β2M) were comparable between the FL2000 and MER cohorts, which justified pooling them to create a larger, independent validation set. They found that their scoring system was also able to accurately predict event-free survival in this independent cohort.

This two-step process of training and validation strengthens the study’s findings and suggests that the simplified scoring system based on LDH and β2m levels could be a valuable tool for clinicians treating patients with de novo follicular lymphoma.

### 2.4. Follicular Lymphoma Evaluation Index

Existing models like FLIPI, FLIPI2 [3], and PRIMA-PI [5] are argued to not be accurate enough in predicting which patients will experience early progression after initial immunochemotherapy. They base this argument on these models’ limited sensitivity, development before modern therapies, and suboptimal sensitivity/specificity [6,7]. The FLIPI has been reported to have a sensitivity of 70–78% but a specificity of only 56–58% for predicting progression or death within 24 months. This means a significant portion of patients deemed high risk by FLIPI might not experience early progression, while some classified as lower risk might progress rapidly. Furthermore, FLIPI and FLIPI2 predate the widespread use of novel agents like obinutuzumab and bendamustine. Their applicability to patients receiving these newer immunochemotherapy regimens may be limited.

To address these limitations, the Follicular Lymphoma Evaluation Index (FLEX) was developed and validated using data from 1202 follicular lymphoma patients treated in the modern therapeutic era. Therefore, 17 potential clinical variables were identified from the GALLIUM trial data based on data availability, prior evidence of association with adverse outcomes, and clinical plausibility. Statistical analysis then narrowed these down to the nine key clinical variables that make up the FLEX score: male sex, splenic involvement with the largest diameter in the highest quartile, histologic grade 3A, more than two extranodal sites, ECOG performance status greater than 1, hemoglobin level less than 12 g/dL, B2M level higher than the institutional upper limit of normal, peripheral blood absolute natural killer cell count less than 100/μL, and LDH level higher than the upper limit of normal. The FLEX score, incorporating these nine variables, was then validated using data from the SABRINA trial. FLEX demonstrated superior predictability for progression of disease within 24 months compared to both FLIPI and FLIPI2. FLEX showed higher specificity for progression of disease within 24 months (POD24) than FLIPI, FLIPI2, and PRIMA-PI, meaning it was better at correctly identifying patients who would not experience early progression. FLEX showed better discrimination ability in predicting progression-free survival at 3 years compared to the other models, as evidenced by the receiver operating characteristic curves [7]. The study found that FLEX was better at identifying high-risk patients than existing models, accurately predicting both progression-free survival and early progression within 24 months. This improved accuracy could help treating physicians personalize treatment plans and potentially improve outcomes for patients with follicular lymphoma.

### 2.5. Progression of Disease Within 24 Months (POD24)

Casulo et al. [8] analyzed data from two independent cohorts of patients with follicular lymphoma treated with R-CHOP. They compared the OS of patients who relapsed within 2 years of diagnosis (early relapse) to those who remained in remission beyond 2 years. To ensure the validity of their findings, they adjusted for other clinical factors that could potentially influence survival. These factors included age: patient age at diagnosis, Ann Arbor stage: the stage of follicular lymphoma at diagnosis (ranging from I to IV, indicating the extent of the disease), And Eastern Cooperative Oncology Group Scale performance status: an assessment of the patient’s overall health and ability to perform daily activities [9]. By including these factors in the multivariate model, the effect of early relapse (progression of disease within 2 years) on overall survival could have been isolated while accounting for the potential confounding influence of these other variables. This approach strengthens the validity of their findings by demonstrating that the association between early relapse and poorer survival is independent of these other known prognostic factors. The analysis revealed a statistically significant difference in survival between the two groups. The authors found that patients who experienced early relapse (within 2 years) had a 5-year overall survival rate of approximately 50%. In contrast, those who remained in remission beyond 2 years had a significantly higher 5-year overall survival rate of about 80%. This difference in survival persisted even after adjusting for the clinical factors mentioned above, strengthening the conclusion that early relapse is a strong independent predictor of poor survival in this patient population.

### 2.6. POD24 Prognostic Index

Jurinovic et al. [6] examines how well clinicogenetic risk models can predict early disease progression in follicular lymphoma patients after initial immunochemotherapy. The study specifically focuses on the prognostic significance of disease progression within 24 months of treatment.

Two independent groups of follicular lymphoma patients, one from the German Low-Grade Lymphoma Study Group and the other from the British Columbia Cancer Agency, were analyzed. The goal was to determine whether established clinicogenetic risk models, such as the Follicular Lymphoma International Prognostic Index, its updated version, and the m7-FLIPI, could predict POD24, a known risk factor for unfavorable outcomes.

The study found that a substantial percentage of patients (15–23% depending on the cohort and calculation method) experienced progression of disease within 24 months (POD24). Importantly, patients with POD24 had significantly worse overall survival compared to those without early progression.

The analysis revealed that all three models (FLIPI, FLIPI2, m7-FLIPI) effectively identified patients at a higher risk of early progression. This suggests that these models can be valuable tools for clinicians to identify patients who might benefit from closer monitoring or alternative treatment strategies.

Furthermore, the study highlighted that even within specific risk groups defined by these models, the presence or absence of POD24 remained a significant factor influencing overall survival. This emphasizes the importance of POD24 as an independent prognostic factor in follicular lymphoma.

In conclusion, the study underscores the value of clinicogenetic risk models like FLIPI, FLIPI2, and m7-FLIPI in predicting early progression of follicular lymphoma after first-line immunochemotherapy. The findings highlight the clinical significance of POD24 as a strong predictor of poor outcomes and emphasize the need for personalized treatment approaches based on individual risk stratification.

### 2.7. FLIPI24

A recent study from an ASH (American Society of Hematology) meeting [10] investigated the FLIPI24 prognostic model, specifically focusing on its ability to predict outcomes for follicular lymphoma patients who did not receive immunochemotherapy as their initial treatment. The patients in this study received non-immunochemotherapy treatments, specifically observation (closely monitoring the lymphoma without immediate active treatment), rituximab monotherapy (treatment with the monoclonal antibody rituximab alone, without chemotherapy drugs), or radiation (using radiation therapy to target and destroy lymphoma cells). Data from 1617 patients were analyzed, comparing FLIPI24 scores with outcomes following these non-immunochemotherapy (IC) approaches. The study found that high FLIPI24 scores correlated with significantly shorter event-free survival and overall survival (*p* < 0.001), even in the absence of initial IC. Additionally, FLIPI24 demonstrated better concordance for OS compared to the original FLIPI in this non-IC group. FLIPI, the original Follicular Lymphoma International Prognostic Index, was designed to predict survival in the pre-rituximab era. FLIPI2, a refined version of FLIPI, incorporated factors relevant to the rituximab era and aimed for improved accuracy. FLIPI24 was developed to predict events (like disease progression or need for treatment) within 24 months of starting IC. This study suggests FLIPI24’s value extends to those receiving non-IC approaches. Even without IC, patients with high FLIPI24 scores experience poorer outcomes, emphasizing the need for closer monitoring or consideration of alternative therapies. This research reinforces the ongoing efforts to refine prognostication in follicular lymphoma, moving beyond the limitations of earlier models like FLIPI. FLIPI24 was using factors like age (linear from 60 to 90 years, with an inflection point at age 75), hemoglobin (linear from 8 to 17 g/dL), white blood cell count (linear from 4 to 11 × 10^9^/L), lactate dehydrogenase/upper limit of normal (linear from 0.5 to 5), and beta-2 microglobulin (B2M, linear from 1 to 10 mg/L). Each of these factors is measured and contributes to a patient’s FLIPI24 score, which helps predict their risk of experiencing events like disease progression or the need for treatment within 24 months. In conclusion, this study demonstrates the utility of FLIPI24 as a prognostic tool even in the absence of initial immunochemotherapy, potentially aiding treatment decisions for a wider range of follicular lymphoma patients.

A comparison of the FLIPI, FLIPI2, PRIMA-PI, FLEX, and FLIPI24 is highlighted in Table 1.

While all these indices aim to predict outcomes in follicular lymphoma, they differ in the specific parameters used, the number of risk groups, and the outcomes they predict. FLIPI and FLIPI2 focus on overall survival, while PRIMA-PI, FLEX, and FLIPI24 consider progression-free survival and other endpoints relevant to treatment response and disease progression. The choice of which index to use depends on the specific clinical context and the information needed for patient management.

## 3. Molecular Markers and Follicular Lymphoma Prognosis

A systematic review [11] analyzed 283 original papers published from January 1984 to September 2024, identified via a PubMed search using PRISMA guidelines. Biomarkers were categorized based on their reported impact on prognosis or transformation risk (none, favorable, or inferior).

Genetic Abnormalities: This category encompasses chromosomal translocations like the characteristic t(14; 18), copy number variations, and specific gene mutations. While t(14; 18) is a hallmark of follicular lymphoma, its predictive power for transformation is debated. Mutations in genes like EZH2, ARID1A, and TP53 are linked to poorer prognoses. CNVs, representing gains or losses of chromosomal segments, can also influence gene expression and clinical outcomes.

Gene Expression: This involves analyzing patterns of gene expression correlated with transformation risk or prognosis. Techniques like microarray analysis and RNA sequencing identify gene signatures. Overexpression of cell cycle or DNA replication genes may signal higher transformation risk, while downregulation of immune regulation or apoptosis genes might be seen in aggressive follicular lymphoma.

MicroRNAs: These small non-coding RNAs regulate gene expression and play a crucial role in cancer. Some miRNAs act as oncogenes, promoting cell growth, while others function as tumor suppressors. Dysregulation of these miRNAs can contribute to follicular lymphoma progression and transformation.

B-Cell/Follicular Lymphoma Tumor Cell Markers: These include surface markers like CD10, CD20, and immunoglobulin expression. While vital for diagnosis, their prognostic value for transformation is limited.

Tumor Microenvironment Markers: The tumor microenvironment is critical in follicular lymphoma progression. Markers reflecting immune cell infiltration (e.g., T cells, macrophages), angiogenesis (e.g., VEGF), and stromal composition are studied. An immunosuppressive TME, rich in regulatory T cells or myeloid-derived suppressor cells, may be associated with worse outcomes.

Soluble Biomarkers: These are circulating factors in blood or serum, such as cytokines, chemokines, and soluble receptors. Elevated levels of certain cytokines may indicate increased inflammation and a more aggressive disease course.

### m7-FLIPI

The incorporation of genetic mutation data was investigated in terms of whether they can enhance the accuracy of risk prediction for follicular lymphoma patients undergoing first-line immunochemotherapy.

A retrospective analysis of a prospective clinical trial was conducted involving 151 follicular lymphoma patients enrolled in the German Low-Grade Lymphoma Study Group 2000 trial. They performed deep sequencing on tumor biopsy samples to analyze mutations in 74 genes. These 74 genes were pre-selected based on their known recurrence in follicular lymphoma and potential relevance to its pathogenesis. The researchers then used multivariable L1-penalized Cox regression to develop a novel prognostic model, termed m7-FLIPI, by integrating these mutations with clinical risk factors. The seven mutations included in the final m7-FLIPI model (EZH2, ARID1A, MEF2B, EP300, FOXO1, CREBBP, and CARD11) were selected because they emerged as the most significant predictors of failure-free survival in this analysis.

The M7-FLIPI model demonstrated superior prognostic accuracy compared to the FLIPI index alone, as evidenced by a higher C-index (0.79 vs. 0.65). Notably, the m7-FLIPI model effectively stratified patients into distinct risk groups with significantly different 5-year failure-free survival rates.

To validate their findings, the researchers applied the m7-FLIPI model to an independent cohort of 546 patients from the British Columbia Cancer Agency Lymphoid Cancer Database. The M7-FLIPI model maintained its prognostic value in this validation cohort, outperforming the FLIPI index and other established prognostic models.

The study’s findings suggest that integrating genetic mutation data with clinical factors can significantly improve risk stratification for follicular lymphoma patients receiving first-line immunochemotherapy. The m7-FLIPI model shows promise as a valuable tool for identifying patients at high risk of treatment failure, potentially guiding personalized treatment decisions and improving clinical outcomes [12].

## 4. Histological Characteristics and Follicular Lymphoma Prognosis

### 4.1. EZH2

EZH2 is a histone methyltransferase that has been implicated in the pathogenesis of follicular lymphoma. Genetic alterations in EZH2 have been associated with a more aggressive clinical course [13]. In particular, high EZH2 expression has been linked to an increased proliferation index and transformation to high-grade disease [14]. A study investigating the prognostic significance of EZH2 mutations in follicular lymphoma found that patients with mutated EZH2 had significantly shorter progression-free survival compared to those with wild-type EZH2 [13].

### 4.2. Ki-67

The relationship between Ki-67 expression, a marker of cell proliferation, and early disease progression in follicular lymphoma was investigated by a Japanese group recently [15]. A cohort of 47 patients diagnosed with follicular lymphoma was analyzed. It was discovered through multivariate analysis that increased Ki-67 expression was significantly associated with a higher risk of disease progression within 24 months. These findings suggest that Ki-67 expression could serve as a valuable prognostic biomarker for identifying follicular lymphoma patients at higher risk of early disease progression. Furthermore, the study highlights the potential role of the tumor microenvironment in influencing follicular lymphoma progression.

### 4.3. Proteomics

A recent study [16] with high-throughput mass spectrometry was employed to analyze protein profiles within diagnostic lymphoma tissue samples from 48 patients diagnosed with advanced-stage follicular lymphoma. The goal was to identify potential protein biomarkers associated with disease progression. A total of 99 proteins were found to be significantly differentially expressed between samples from patients who experienced subsequent progression (sp-FL) and those who did not (np-FL). Using these proteins, patients were classified into high-risk and low-risk subgroups using unsupervised machine learning techniques. Pathway analyses of the identified proteins revealed potential disruptions within the immune system and cellular energy metabolism. Two proteins, STING1 and IDH2, were selected for further immunohistochemical evaluation based on their differential expression and potential roles in follicular lymphoma biology. Notably, IDH2 expression was significantly associated with progression-free survival. This study highlights the potential of proteomic profiling to identify novel biomarkers and therapeutic targets for follicular lymphoma, potentially leading to improved risk stratification and personalized treatment strategies.

## 5. PET/CT as Prognostic Marker in Follicular Lymphoma

### 5.1. Staging and Follicular Lymphoma Prognosis

Precise staging is crucial for follicular lymphoma prognosis, as it directly informs treatment strategies and predicts patient outcomes. The Ann Arbor staging system, combined with modern imaging techniques like computed tomography and positron emission tomography, is the standard for assessing disease extent [17]. Positron emission tomography (PET) scans, in particular, have improved staging accuracy by detecting involvement in small nodes and extranodal sites often missed by computed tomography (CT) alone [18]. While higher Ann Arbor stages generally correlate with a worse prognosis, the relationship is multifaceted and influenced by additional factors like histological grade, genetic features, and treatment response. Furthermore, the use of PET scans for staging has been associated with improved overall survival, likely due to more accurate risk stratification and subsequent treatment optimization [18]. Therefore, accurate staging is not only essential for classifying disease extent but also for tailoring treatment approaches and improving patient outcomes in follicular lymphoma.

Li et al. [19] explored the prognostic value of baseline PET/CT parameters in follicular lymphoma, including a novel measure called Dmax (distance between the two furthest lesions). Their retrospective analysis of 126 patients revealed that higher Dmax, indicative of more widespread disease, correlated significantly with poorer progression-free survival. Similarly, increased total metabolic tumor volume and total lesion glycolysis, reflecting a larger and more metabolically active tumor burden, were linked to worse PFS. While the study’s primary focus was on prognostication rather than staging, it briefly touched upon the role of SUVmax. Although previous research has suggested SUVmax can differentiate between grade 1/2 and grade 3A follicular lymphoma, Li et al. did not observe this association in their cohort. Importantly, SUVmax, while a useful marker of metabolic activity, is not a primary factor in formal Ann Arbor staging, which emphasizes anatomical extent of disease. Therefore, Li et al. highlights the potential of PET-derived metrics, particularly Dmax, to refine prognostic assessments in follicular lymphoma beyond traditional staging methods.

Draye-Carbonnier et al. [20] conducted a retrospective study on 126 patients with high-burden follicular lymphoma (grades 1–3a) to evaluate the prognostic value of quantitative features extracted from baseline PET/CT scans. They used Oncometer3D software (version 1.043) to measure parameters related to tumor volume and fragmentation. Specifically, total metabolic tumor volume, tumor volume spherical ratio, and median probabilistic distance (medPCD, a measure of tumor massiveness) were identified as independent predictors of progression of disease at 24 months. A combined prognostic score incorporating these three parameters demonstrated superior predictive performance compared to using the parameters individually. The study highlights the potential of these PET/CT-derived features to improve risk stratification and guide personalized treatment decisions in patients with high-burden follicular lymphoma. The authors suggest that this approach could identify patients at higher risk of early progression who might benefit from more intensive or alternative treatment strategies.

Our working group [21] retrospectively analyzed data from 143 follicular lymphoma patients to investigate prognostic factors influencing survival. They found that a maximum standardized uptake value cutoff of 9.85 on staging PET/CT and a lymphocyte/monocyte (Ly/Mo) ratio of 3.41 at diagnosis were significant predictors of progression-free survival. Combining both factors further stratified patients into high- and low-risk groups. The last three studies investigate prognostic factors in follicular lymphoma using baseline data, but they employ different parameters. Ref. [21] uses SUVmax and Ly/Mo ratio, Ref. [20] focuses on volumetric and spatial PET parameters, and Ref. [19] introduces Dmax (distance between furthest lesions) alongside standard metabolic parameters. All three utilize PET/CT, but Ref. [21] incorporates a readily available blood biomarker (Ly/Mo) in conjunction with PET data. While all studies include follicular lymphoma patients, Ref. [20] specifically focuses on high-tumor-burden follicular lymphoma, while Refs. [19,21] include broader follicular lymphoma populations. Ref. [21] examines PFS, while [20] focuses on progression of disease at 24 months, and Ref. [19] primarily assesses PFS. Table 2 summarizes the key features of the three studies, highlighting their respective patient populations, study foci, and main findings related to PET/CT parameters and prognostication in follicular lymphoma.

This table summarizes the key features of the three studies, highlighting their respective patient populations, study foci, and main findings related to PET/CT parameters and prognostication in follicular lymphoma. Draye-Carbonnier et al. focuses specifically on high-tumor-burden follicular lymphoma, while Li et al. also explores the use of PET/CT for grading. Jóna et al. investigates a broader range of prognostic factors, including clinical parameters like the lymphocyte/monocyte ratio [20,21].

### 5.2. Total Metabolic Tumor Volume

The Relevance trial was a phase III study comparing lenalidomide plus rituximab (R2) versus R-chemotherapy followed by rituximab maintenance in patients with previously untreated advanced-stage follicular lymphoma. As part of the trial, researchers conducted an analysis to determine the prognostic value of baseline PET parameters—specifically, total metabolic tumor volume (TMTV) [22]. Of the 1032 patients enrolled, 406 were evaluable by PET at baseline. TMTV was calculated using a 41% SUVmax thresholding method. With a median follow-up of 6.5 years, the 6-year progression-free survival rate was 57.8%. The median TMTV was 284 cm^3^, the median SUVmax was 11.3, and the median standardized maximal distance between two lesions was 10.5 cm. The study found that a TMTV > 510 cm^3^ was significantly associated with shorter PFS. Patients with TMTV ≤ 510 cm^3^ had a 3-year PFS of 85.1%, while those with TMTV > 510 cm^3^ had a 3-year PFS of 77.3% (hazard ratio 1.59, *p* < 0.013). This suggests that baseline TMTV can provide valuable prognostic information in advanced-stage follicular lymphoma, even in the context of modern chemo-immunotherapy regimens. The study also highlighted the potential of automated TMTV calculation methods based on deep learning algorithms, which showed excellent correlation with manually calculated TMTV and could facilitate the incorporation of this parameter into routine clinical practice.

A retrospective, multicenter study [23] investigated the prognostic value of TMTV in patients with initially observed, low-tumor-burden follicular lymphoma. The study included 97 patients with stage III–IV follicular lymphoma, all of whom were initially managed with watchful waiting due to their low-tumor-burden status. These patients met specific criteria for low tumor burden, including no B symptoms, no bulky disease, and no other high-risk features. The median age was 61 years, and the majority had grade 1–2 histology. Baseline PET/CT scans were performed for all patients, and TMTV was calculated using a standardized method. The median TMTV was 138 mL, with a range from 0 to over 3000 mL. A TMTV cutoff of 50 mL was used to stratify patients into high- and low-TMTV groups. The primary endpoint was time to first treatment. Patients with a high TMTV (>50 mL) had a significantly shorter time to first treatment (TTFT) compared to those with a low TMTV (median 2.6 years vs. 8.8 years, *p* = 0.001). The 5-year probability of requiring treatment was 77% in the high-TMTV group and 46% in the low-TMTV group. All patients who initiated treatment did so due to disease progression—most commonly, lymph node enlargement. In multivariable analysis, which included factors like age, stage, FLIPI score, and other PET parameters, only high TMTV remained an independent predictor of shorter TTFT (hazard ratio 3.6, 95% CI 1.8–7.3, *p* = 0.001). The authors concluded that TMTV is a strong and independent predictor of TTFT in advanced-stage, low-tumor-burden follicular lymphoma managed with watchful waiting. They suggested that TMTV, readily obtainable from standard PET/CT scans, could help identify patients who might benefit from earlier intervention, although further prospective validation is needed.

Another retrospective study [20] investigated the prognostic value of combining volume, massiveness, and fragmentation parameters measured on baseline FDG PET/CT in patients with high-burden follicular lymphoma. Researchers analyzed data from 126 patients diagnosed between 2006 and 2020. Using Oncometer3D software, they extracted several PET/CT-derived features, including total metabolic tumor volume, tumor volume spherical ratio, and median probabilistic distance (medPCD, a measure of fragmentation). They found that TMTV, tumor-volume-to-spleen-volume ratio (TVSR), and medPCD were independent predictors of 24-month progression of disease. A combined prognostic score incorporating these three parameters showed superior predictive performance compared to using them individually. Patients with all three parameters at high levels had significantly worse outcomes (hazard ratio 11.2, *p* < 0.001) compared to other risk groups. The study suggests that combining these PET-derived parameters can improve risk stratification and potentially guide treatment decisions in high-burden follicular lymphoma.

The key differences between the three studies lie in their patient populations and the specific outcomes examined. Cottereau et al. [22] focused on advanced-stage follicular lymphoma patients within a clinical trial setting, examining progression-free survival. Mozas et al. [23] specifically studied patients with low-tumor-burden advanced-stage follicular lymphoma initially managed with watchful waiting, with time to first treatment as their outcome. Draye-Carbonnier et al. [20] investigated high-tumor-burden follicular lymphoma and looked at 24-month progression of disease, also incorporating additional PET parameters like tumor volume spherical ratio and median probabilistic distance.

The common thread is the use of TMTV as a prognostic factor. All three studies demonstrated its value, albeit in different contexts and with varying cutoff values. While Cottereau et al. [22] used a TMTV cutoff of 510 cm^3^ to predict PFS, Mozas et al. [23] used a much lower cutoff of 50 mL to predict TTFT. This difference likely reflects the distinct patient populations and the nature of the outcomes being studied. Draye-Carbonnier et al. [20] further emphasized the potential of combining TMTV with other PET-derived parameters to refine prognostication, particularly in high-tumor-burden follicular lymphoma.

### 5.3. Treatment Response and Follicular Lymphoma Prognosis

The ability to predict and monitor treatment response is another critical aspect of follicular lymphoma prognosis. Luminari et al. [24] analyzes the impact of end-of-treatment PET response and minimal residual disease on progression-free survival in follicular lymphoma patients. It is a subset analysis from the FOLL05 trial, which randomized patients to R-CVP, R-CHOP, or R-FM. The study found that both restaging PET negativity and minimal residual disease (MRD) negativity were independently associated with improved PFS. Combined PET and MRD assessment further refined prognostication, identifying a group with excellent PFS. Ref. [25] is a secondary analysis of the GALLIUM trial, investigating the prognostic value of end-of-induction PET response in follicular lymphoma patients treated with first-line immunochemotherapy (obinutuzumab or rituximab plus maintenance). The study confirmed that achieving a complete metabolic response on PET was strongly associated with improved PFS and overall survival. Both studies highlight the prognostic significance of PET response in follicular lymphoma. Ref. [24] focuses on end-of-treatment PET and incorporates MRD assessment, while Ref. [25] examines end-of-induction PET in the context of modern immunochemotherapy regimens.

### 5.4. Relapse and Follicular Lymphoma Prognosis

Lee et al. [26] investigates survival outcomes of relapsed/refractory follicular lymphoma patients, emphasizing the difficulties in their management and exploring prognostic factors. The study found poor overall survival and frequent relapse even after standard salvage chemotherapy, especially in patients with relapse/progression within 24 months. Autologous stem cell transplantation after first relapse showed potential for prolonging survival, particularly in POD24 patients, suggesting its value as a consolidation approach. Participation in clinical trials was associated with improved survival trends, highlighting the importance of enrolling these patients in trials for access to novel therapies. The study underscores the variability in clinical courses and treatment responses, emphasizing the need for personalized treatment based on individual factors and risk. Overall, Ref. [26] reinforces the challenges in treating relapsed/refractory follicular lymphoma and suggests ASCT and clinical trial participation as potential avenues for improved survival, especially in patients with early relapse/progression. It also highlights the need for further research and personalized strategies to address the heterogeneous outcomes in this group.

## 6. Transformation and Follicular Lymphoma Prognosis

Vaughn et al. [27] is a population-based study using the Surveillance, Epidemiology, and End Results-18 database, comparing the survival of patients with transformed follicular lymphoma (t-FL) and de novo diffuse large B-cell lymphoma (DLBCL) diagnosed in the US between 2010 and 2018. The SEER program collects and publishes cancer incidence and survival data from population-based cancer registries covering approximately 35% of the US population. The study included 569 cases of t-FL and 44,706 cases of de novo DLBCL. The study aimed to determine whether t-FL patients have inferior survival compared to de novo DLBCL patients. They analyzed relative survival, overall survival, and lymphoma-specific survival. The results showed that t-FL patients had significantly lower survival rates across all measures. The 5-year RS for t-FL was 54% compared to 67% for de novo DLBCL. The 5-year OS was 49% for t-FL and 57% for de novo DLBCL. The 5-year lymphoma-specific survival (LSS) was 54% for t-FL and 66% for de novo DLBCL. These differences were statistically significant. The study concludes that t-FL continues to have a worse prognosis than de novo DLBCL, even with recent treatment advancements, and recommends prioritizing t-FL patients for clinical trial enrollment to explore new treatment strategies. The study is presented as a letter to the editor.

An American Society of Hematology (ASH) abstract [28] analyzes prognostic factors and outcomes of transformed follicular lymphoma (t-FL) in 306 patients identified between 2002 and 2021 from the Lymphoma Epidemiology of Outcomes Consortium. The study found that t-FL carries a poor prognosis, with reported median overall survival ranging from 22 to 50 months. Several factors were found to influence survival after transformation. A shorter time from initial follicular lymphoma diagnosis to transformation, specifically early transformation (within 24 months), is linked to worse outcomes. A total of 69% of transformations after immunochemotherapy occurred within 24 months. A higher International Prognostic Index score at the time of transformation also indicated a poorer prognosis. Prior exposure to anthracyclines did not significantly impact survival after transformation. However, the study suggests that more intensive first-line therapy and the use of autologous stem cell transplantation may improve outcomes. The study concludes that t-FL remains a clinically challenging disease with variable outcomes. Identifying these prognostic factors, such as time to transformation and IPI score, can help stratify patients by risk and guide treatment decisions. Further research is needed to refine treatment strategies and improve survival in t-FL. The study reinforces the findings of [27] regarding the worse prognosis of t-FL and highlights the importance of identifying prognostic factors like time to transformation and IPI for risk stratification and treatment guidance.

Kalashnikov et al. [29] conducted a Finnish nationwide population-based study examining survival, transformation, and causes of death in follicular lymphoma patients diagnosed between 1995 and 2018. The study specifically highlights that a substantial proportion of follicular lymphoma transform to large B-cell lymphoma, leading to a worse prognosis. The 10-year risk of transformation was found to be 8.4%. Transformation was significantly associated with increased mortality (HR 5.01; 95% CI, 4.21–5.96). In addition, follicular lymphoma grade 3A itself was also associated with a higher risk of death (HR 1.42; 95% CI, 1.13–1.78) compared to lower grades. The study underscores the importance of transformation as a key event impacting survival in follicular lymphoma and notes that lymphoma, including transformed follicular lymphoma, remained the leading cause of death in these patients. Comparing this with [26,28], [29]’s 8.4% 10-year transformation risk aligns with [28]’s findings of infrequent transformation (around 2% per year in the rituximab era). Ref. [26] does not explicitly state an overall transformation rate but notes transformation in a subset of patients with early progression. All three studies highlight the negative impact of transformation on survival. Ref. [28] focuses on prognostic factors after transformation (time to transformation, IPI), while Ref. [29] emphasizes the impact of transformation itself and follicular lymphoma grade. Ref. [26] considers time to progression as a key factor, which indirectly relates to transformation risk. Ref. [29] is a population-based study, providing a broader perspective than the multi-center cohort study of Ref. [28] or the single-center analysis of Ref. [26]. This difference in design influences the generalizability of the findings. In summary, all three studies underscore the importance of transformation as a critical event in follicular lymphoma prognosis. Ref. [29] provides a population-level perspective on transformation risk, while Refs. [26,28] offer more granular insights into prognostic factors and outcomes within specific patient cohorts.

## 7. Patient Factors and Follicular Lymphoma Prognosis

Our group’s research has highlighted the prognostic significance of the lymphocyte/monocyte (Ly/Mo) ratio and age in follicular lymphoma. In a retrospective study of 143 follicular lymphoma patients [21], we found that a Ly/Mo ratio of 3.41 at diagnosis significantly predicted progression-free survival. Furthermore, combining a high maximum standardized uptake value (SUVmax > 9.85) on staging PET/CT with a low Ly/Mo ratio (<3.41) identified a high-risk group with significantly worse PFS. A separate study [30] focusing on 49 follicular lymphoma patients undergoing autologous stem cell transplantation revealed that both age and the Ly/Mo ratio were independent predictors of PFS after ASCT. Specifically, patients older than 47 years with a pre-transplant Ly/Mo ratio ≥ 2.675 experienced significantly worse PFS, indicating a high-risk profile for this subgroup. These findings suggest that readily available clinical parameters like the Ly/Mo ratio and age, especially in combination with PET/CT findings, can effectively risk-stratify follicular lymphoma patients and inform treatment decisions, particularly in the context of autologous stem cell transplantation.

The Ly/Mo ratio is an emerging prognostic factor in several cancers, including follicular lymphoma [31], reflecting the complex interplay between the immune system and the tumor microenvironment. A higher Ly/Mo ratio generally suggests a more favorable prognosis, while a lower Ly/Mo ratio is often associated with worse outcomes. This is because lymphocytes, particularly cytotoxic T cells, play a crucial role in anti-tumor immunity, while monocytes can differentiate into tumor-associated macrophages (TAMs) within the tumor microenvironment. TAMs are a heterogeneous population of immune cells that can exert both pro- and anti-tumor effects. In many cancers, however, TAMs are predominantly associated with tumor progression, angiogenesis, metastasis, and immunosuppression. They can suppress T-cell activity, promote tumor growth, and contribute to treatment resistance. Therefore, a lower Ly/Mo ratio, indicating a relative abundance of monocytes compared to lymphocytes, may reflect a more immunosuppressive tumor microenvironment that favors tumor growth and spread [21].

It is important to note that the Ly/Mo ratio is just one factor. The tumor microenvironment is a complex ecosystem involving various cell types, signaling molecules, and extracellular matrix components. While Ly/Mo ratio can provide valuable prognostic information, it should be considered in conjunction with other clinical and biological factors for a comprehensive assessment of disease status and treatment planning.

## 8. Summary

Follicular lymphoma is an indolent yet incurable B-cell malignancy with a variable clinical course. Recent studies have provided important insights into factors influencing prognosis and survival in follicular lymphoma patients. While follicular lymphoma remains an incurable disease, advances in immunochemotherapy and the development of novel targeted therapies have led to improved overall survival outcomes, which could be further enhanced by risk-adapted treatment strategies [32] and incorporating prognostic factors like clinical parameters, molecular markers, PET/CT, and transformation status of follicular lymphoma patients.

For routine clinical practice in follicular lymphoma, the most useful prognostic tools balance predictive accuracy with ease of use and accessibility. At diagnosis, FLIPI remains a cornerstone for initial risk stratification. While not perfect, it is simple to calculate and provides a good starting point for risk assessment [1,3]. FLIPI2, the updated version, incorporates beta2-microglobulin and the size of the largest involved node, offering improved prognostication compared to FLIPI. However, obtaining beta2-microglobulin adds complexity, and its added benefit might not outweigh the extra step in routine practice [1,3].

In terms of evaluation during and post treatment, interim and end-of-treatment PET/CT scans are invaluable for assessing response to therapy. Qualitative assessments (e.g., Deauville score) are widely used and readily interpretable. Quantitative measures like total metabolic tumor volume and total lesion glycolysis offer more refined prognostic information but require specialized software and expertise, limiting their routine use [19]. Monitoring for transformation to diffuse large B-cell lymphoma is crucial, as it significantly impacts prognosis. Regular clinical follow-up and consideration of biopsy for suspected transformation are essential.

Less commonly used in routine practice is the m7-FLIPI, which incorporates mutational data, adding significant prognostic value. However, routine mutational testing is not yet standard practice due to cost and complexity. The PRIMA-PI is designed for patients receiving immunochemotherapy; this index uses beta2-microglobulin and bone marrow involvement. While useful in specific settings, it is not as widely adopted as FLIPI or FLIPI2 [19]. The Ly/Mo ratio suggests its prognostic value, particularly when combined with PET/CT findings [21,31], but further validation is needed before routine clinical adoption.

**Expert Recommendation:** In everyday practice, we recommend starting with FLIPI at diagnosis for initial risk stratification. PET/CT-based assessment should be incorporated during and after treatment to monitor response and guide decisions. It is important to remain vigilant for transformation, as it significantly alters prognosis. FLIPI2 should be considered if beta2-microglobulin is readily available, but its added benefit in routine practice might be limited. While newer tools like m7-FLIPI and Ly/Mo ratio show promise, they require further validation before becoming standard practice. Ultimately, the choice of prognostic tools should be guided by clinical context, resource availability, and patient-specific factors.

## Figures and Tables

**Table 1:** Clinical prognostic indices.

Feature	FLIPI [2]	FLIPI2 [3]	PRIMA-PI [5]	FLEX [7]	FLIPI24 [10]
**Parameters**	Age > 60, Ann Arbor stage III/IV, hemoglobin < 12 g/dL, number of nodal areas > 4, serum LDH > normal	β2-microglobulin, number of nodal areas > 4, age > 60, hemoglobin < 12 g/dL, serum LDH > normal	β2-microglobulin > 3 mg/L, Ann Arbor stage III/IV, hemoglobin < 12 g/dL	Age > 60, Ann Arbor stage III/IV, ECOG PS ≥ 2, Hb < 12 g/dL, ≥1 extranodal site, B-symptoms, LDH > ULN, β2M > 3 mg/L, GCB/non-GCB subtype	Age (linear 60–90 years with inflection at age 75), HGB (linear 8–17 g/dL), WBC (linear 4–11 × 10^9^/L), LDH/ULN (linear 0.5–5), and B2M (linear 1–10 mg/L)
**Risk Groups**	Low, intermediate, high	Low, intermediate, high	Low, intermediate, high	Low, high	Low, low–average, average, high, very high
**Outcome Measured**	Overall survival	Overall survival	Progression-free survival at 24 months	Progression-free survival, progression of disease within 24 months, overall survival	Event-free survival at 24 months, overall survival

**Table 2:** Staging PET/CT.

Feature	Li et al. (2022) [19]	Draye-Carbonnier et al. (2024) [20]	Jóna et al. (2022) [21]
**Patient Population**	126 patients with grade 1–3A follicular lymphoma	105 follicular lymphoma patients with high tumor burden	143 follicular lymphoma patients
**Study Focus**	Predicting progression-free survival and pathologic grade using PET/CT parameters	Evaluating the prognostic value of PET/CT parameters in high-tumor-burden FL	Investigating prognostic factors influencing survival, including SUVmax and lymphocyte/monocyte ratio
**Key Findings**	High Dmax, TLG, and LDH are independent prognostic factors for PFS. A novel scoring system based on these parameters showed superior performance. PET/CT may help distinguish grade 3A from low-grade FL.	Combination of high TMTV, TVSR, and medPCD significantly predicted shorter PFS in high tumor burden FL.	SUVmax on staging PET/CT and lymphocyte/monocyte ratio at diagnosis significantly predicted PFS. Combination of high SUVmax and low Ly/Mo ratio identified a high-risk group.
**PET/CT Parameters**	Dmax (distance between furthest lesions), TLG (total lesion glycolysis), SUVmax	TMTV (total metabolic tumor volume), TVSR (tumor volume to spleen volume ratio), medPCD (median of the perpendicular component of the metabolic tumor diameter)	SUVmax (staging, interim, and restaging)

## Data Availability

No data of human subjects were involved in this manuscript.
